# Psychological distress is highly prevalent in inflammatory bowel disease: A survey of psychological needs and attitudes

**DOI:** 10.1002/jgh3.12236

**Published:** 2019-08-02

**Authors:** Antonina Mikocka‐Walus, Wayne Massuger, Simon R Knowles, Gregory T Moore, Stephanie Buckton, William Connell, Paul Pavli, Leanne Raven, Jane M Andrews

**Affiliations:** ^1^ School of Psychology Deakin University Melbourne Victoria Australia; ^2^ Crohn's & Colitis Australia Melbourne Victoria Australia; ^3^ School of Health Sciences Swinburne University of Technology Melbourne Victoria Australia; ^4^ Department of Gastroenterology Monash Medical Centre Melbourne Victoria Australia; ^5^ School of Clinical Sciences Monash University Melbourne Victoria Australia; ^6^ Department of Gastroenterology Sunshine Coast University Hospital Sunshine Coast Queensland Australia; ^7^ Department of Gastroenterology St Vincent Hospital Melbourne Victoria Australia; ^8^ Department of Gastroenterology Canberra Hospital Canberra Australian Capital Territory Australia; ^9^ Faculty of Science, Health and Engineering University of Sunshine Coast Sippy Downs Queensland Australia; ^10^ IBD Service, Department of Gastroenterology and Hepatology Royal Adelaide Hospital Adelaide South Australia Australia

**Keywords:** distress, inflammatory bowel disease, psychological services

## Abstract

**Background and Aim:**

Data on patient needs and access to psychological services in inflammatory bowel disease (IBD) are scarce. This study aimed to describe the levels of distress and the needs, attitudes, and access to psychological services for people within Australia against established Australian IBD Standards.

**Methods:**

An online cross‐sectional survey was conducted with Australians ≥16 years old recruited via Crohn's & Colitis Australia membership, public and private clinics, and the Royal Flying Doctor Service. K10 was used to measure psychological distress. The Chi‐square test was used to compare those with and without distress on key variables.

**Results:**

Overall, 731 respondents provided complete data (71.5% female, mean age 46.5 years). Overall, 50% of respondents reported distress; only 15.2% were currently seeing a mental health practitioner; only 16.1% were asked about their mental health by their IBD specialist or IBD nurse; and only 12.2% reported access to a mental health practitioner as part of their IBD service. Those with psychological distress were significantly less satisfied with their IBD care; more commonly hospitalized; had an active disease, fistula or perianal disease, pain, or fatigue; and were receiving steroids, opioids, or antidepressants (all *P* < 0.05). As many as 68.2% of those with severe distress were not seeing a mental health practitioner.

**Conclusions:**

The integrated biopsychosocial model of health care, with regular mental health screening and good access to mental health professionals, is requested by people living with IBD to improve their outcomes.

## Graphical abstract

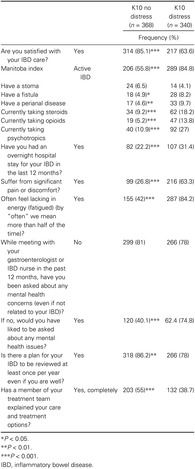

This large Australian survey shows that the integrated biopsychosocial model of health care, with regular mental health screening and good access to mental health professionals, is requested by people living with inflammatory bowel disease to improve their outcomes.

## Introduction

Inflammatory bowel disease (IBD) affects 5 million people around the world, with an estimated 85 000 Australians having Crohn's disease, ulcerative colitis, or indeterminate colitis. People living with IBD suffer from chronic pain, frequent loose bowel movements, weight loss, anemia, and fatigue, significantly impairing their everyday functioning and costing the Australian public $380 million in productivity losses and a further $2.7 billion associated with the management of IBD each year.^1^

IBD is increasingly considered a disorder of the brain–gut axis, where the gut's health is affected by the brain's health and vice versa. For example, it is now well established that, when a person suffers from IBD, they are at a higher risk of developing anxiety and/or depression^2^ and having poorer quality of life^3^, ^4^ than the healthy population. The bidirectional links between mental and physical health in IBD have also been established^5^: Baseline IBD activity is associated with a nearly sixfold increase in the risk of anxiety over a period of 2 years, while anxious patients in IBD remission at baseline have a greater need for steroids, escalation of therapy, or IBD flare over time than those without anxiety. Moreover, the course of IBD is negatively affected by mental illness, resulting in more frequent flare ups,^6^, ^7^ a more aggressive presentation,^8^ hospital readmissions,^9^ and increased risk of surgery.^10^

Despite the high prevalence of mental illness in IBD^2^ and the fact that the International IBD Guidelines^11^ and the Australian IBD Standards (Standard A: High Quality Clinical Care)^12^ recommend regular screening for distress and collaboration with mental health‐care specialists in IBD management, recent studies demonstrated that only a fraction of patients reporting anxiety and depression receive the psychological or psychiatric help they need.^13^, ^14^ Of interest, gender, education, perceived disease activity, and severity of psychological distress did not influence people's likelihood of using mental health services; however, income did, with those with lower income more willing to engage.^14^

Data on patient needs and access to psychological services in IBD are scarce. A national audit of IBD hospital care found a major gap in the access to multidisciplinary services, including psychology support,^15^, ^16^ but the data for mental health services outside inpatient care are lacking. The present survey was designed to describe the levels of distress in people with IBD, as well as the needs, attitudes, and access to psychological services for people with IBD in Australia against established Australian IBD Standards,^12^ which recommend psychological screening for all IBD patients, access to mental health services for inpatient assessment, and treatment and coordination of psychological treatment with primary care mental health services (Standard A13).

## Methods

### *Design*

The data for this study are derived from a large Australia‐wide online survey of quality of health care for people with IBD. This study focused on comparing patient experience of health services with the Australian IBD Standards 2016.^12^ The full methods are presented elsewhere.^17^

### *Participants*

People diagnosed with Crohn's disease, ulcerative colitis, or indeterminate colitis (self‐reported) who were aged 16 years or older were recruited via Crohn's & Colitis Australia membership, public and private medical clinics throughout Australia, and the Royal Flying Doctor Service.

### *Measures*

In addition to the demographics, disease activity, and the quality of care measures previously described,^17^ psychological distress was also measured using the Kessler Psychological Distress Scale (K10). K10^18^ is a simple measure of psychological distress using a 5‐point Likert scale. The maximum score is 50, indicating severe distress; the minimum score is 10, indicating no distress.

### *Analysis*

Data were collected using Qualtrics and analyzed using SPSS Statistics for Windows, Version 25.0; Armonk, NY, IBM Corp, USA. Descriptive statistics included means (SD) and frequencies (%). The Chi‐square test was used to compare those with and without distress on key variables and present mental health engagement by distress level. A *P* value of less than 0.05 was considered significant.

### *Ethical approval*

The study was approved by the Deakin University Human Research Ethics Committee and was performed in accordance with the Declaration of Helsinki. Participants gave informed consent before completing the survey.

## Results

Overall, 731 people provided complete data: 71.5% female, 45.9% university educated, 65.1% married/de facto, 34.1% in full‐time employment, and 57.6% with Crohn's disease. Full demographics and clinical characteristics are presented elsewhere.^17^

### *Psychological care in IBD (Australian IBD Standard A13)*

#### *Level of distress*

The mean level of distress for these respondents (K10 = 20.9) was within the mild distress category (20–24), while 51% of respondents reported good mental health (K10 score < 20), and 15% reported severe distress (K10 score ≥ 30) (Table 1).

**Table 1:** Mental health characteristics and mental health care (Standard A13: Psychological care)

		IBD (*n* = 731)
K10, mean (SD)		20.9 (8.2)
K10†, *n* (%)	Likely well (score 10–19)	369 (50.5)
	Likely mild distress (score 20–24)	135 (18.5)
	Likely moderate distress (score 25–29)	96 (13.1)
	Likely severe distress (score 30–50)	110 (15)
Are you seeing anyone currently for any mental health issues/stress?‡	Yes	111 (15.2)
	Seeing a psychiatrist	19 (2.6)
	Seeing a psychologist	81 (11.1)
	Seeing a general practitioner	45 (6.2)
	Seeing a nurse	1 (0.1)
	Seeing a counselor	8 (1.1)
	Seeing a social worker	2 (0.3)
	Seeing CAM therapist	2 (0.3)
If you are seeing a mental health expert, how often do you meet?	Weekly (of the total of *n* = 111)	9 (8.1)
	Fortnightly (of the total of *n* = 111)	17 (15.3)
	Once a month (of the total of *n* = 111)	36 (32.4)
	Less often than once a month (of the total of *n* = 111)	49 (44.1)
Are you satisfied with the treatment for your mental health issues?	Yes (of the total of *n* = 111)	81 (73)
While meeting with your gastroenterologist or IBD nurse in the past 12 months, have you been asked about any mental health concerns (even if not related to your IBD)?§	Yes	118 (16.1)
If not asked, would you have liked to be asked about any mental health issues?¶	Yes	322 (56)
Do you have access to a mental health expert (i.e. psychologist, psychiatrist) as part of your IBD service?††	Yes	89 (12.2)
I believe having access to a mental health expert (i.e. psychologist, psychiatrist) is an important part of managing my IBD††	Strongly agree	229 (31.3)
	Agree	202 (27.6)
	Neither agree nor disagree	243 (33.2)
	Disagree	34 (4.7)
	Strongly disagree	13 (1.8)
I would like my gastroenterologist or IBD nurse to ask about any mental health concerns (even if not related to my IBD)^††^	Yes	472 (64.6)
I believe accessing mental health services (i.e. psychologist, psychiatrist) is easy to arrange††	Strongly agree	53 (7.3)
	Agree	192 (26.3)
	Neither agree nor disagree	310 (42.4)
	Disagree	128 (17.5)
	Strongly disagree	38 (5.2)
I have found it easy (or would find it easy) to talk to my general practitioner about seeking mental health support††	Strongly agree	180 (24.6)
	Agree	258 (35.3)
	Neither agree nor disagree	193 (26.4)
	Disagree	74 (10.1)
	Strongly disagree	16 (2.2)
I have found it easy (or would find it easy) to talk to my gastroenterologist/IBD nurse about seeking mental health support††	Strongly agree	104 (14.2)
	Agree	212 (29)
	Neither agree nor disagree	252 (34.5)
	Disagree	123 (16.8)
	Strongly disagree	30 (4.1)

† Missing *n* = 21 (2.9%).

‡ Missing *n* = 7 (1%).

§ Missing *n* = 8.

¶ Missing *n* = 156.

†† Missing *n* = 10.

CAM, complementary and alternative medicine; IBD, inflammatory bowel disease.

#### *Mental health practitioners*

Overall, 15.2% of the respondents (*n* = 111) reported currently seeing a mental health practitioner, and for most of those respondents (73%), this was a psychologist (*n* = 81) (Table 1). The most common frequency of visits to a mental health practitioner was less often than once a month. Overall, 73% of those accessing mental health practitioners were satisfied with the treatment received.

#### *Mental health as part of IBD management*

Only 16.1% of respondents were asked about their mental health by their gastroenterologist or IBD nurse, and 56% of those not asked would have liked to be asked. Overall, 64.6% of the respondents would like their gastroenterologist or IBD nurse to ask about their mental health concerns.

Only 12.2% reported access to a mental health practitioner as part of their IBD service, with 58.9% agreeing or strongly agreeing that having access to a mental health expert (i.e. psychologist, psychiatrist) is an important part of managing their IBD. Only 33.6% of respondents believed that accessing mental health services (i.e. psychologist, psychiatrist) was easy to arrange. Nearly 60% would have found it easy to talk to their general practitioner about seeking mental health support, while 43.2% would have found it easy to discuss mental health with a gastroenterologist/IBD nurse.

#### *Poor outcomes are associated with distress*

Those with psychological distress were significantly less satisfied with their IBD care than those not distressed (Table 2). More commonly, participants having distress reported that they had an active disease (using patient‐reported symptom‐based instruments), fistula, or perianal disease and were taking steroids, opioids, or antidepressants (all *P* < 0.05).

**Table 2:** Distress *versus* no distress

		K10 no distress (*n* = 368)	K10 distress (*n* = 340)
		Frequency (%)	
Are you satisfied with your IBD care?	Yes	314 (85.1)***	217 (63.6)
Manitoba index	Active IBD	206 (55.8)***	289 (84.8)
Have a stoma		24 (6.5)	14 (4.1)
Have a fistula		18 (4.9)*	28 (8.2)
Have a perianal disease		17 (4.6)**	33 (9.7)
Currently taking steroids		34 (9.2)***	62 (18.2)
Currently taking opioids		19 (5.2)***	47 (13.8)
Currently taking psychotropics		40 (10.9)***	92 (27)
Have you had an overnight hospital stay for your IBD in the last 12 months?	Yes	82 (22.2)***	107 (31.4)
Suffer from significant pain or discomfort?	Yes	99 (26.8)***	216 (63.3)
Often feel lacking in energy (fatigued) (by “often” we mean more than half of the time)?	Yes	155 (42)***	287 (84.2)
While meeting with your gastroenterologist or IBD nurse in the past 12 months, have you been asked about any mental health concerns (even if not related to your IBD)?	No	299 (81)	266 (78)
If no, would you have liked to be asked about any mental health issues?	Yes	120 (40.1)***	62.4 (74.8)
Is there a plan for your IBD to be reviewed at least once per year even if you are well?	Yes	318 (86.2)**	266 (78)
Has a member of your treatment team explained your care and treatment options?	Yes, completely	203 (55)***	132 (38.7)

* *P* < 0.05.

** *P* < 0.01.

*** *P* < 0.001.

IBD, inflammatory bowel disease.

Those distressed more commonly reported having been hospitalized in the previous year for their IBD and had more pain or fatigue. Those distressed would have liked to have been asked about their mental health by their gastroenterology team, less commonly had a plan of their IBD being reviewed annually, or reported that their care was explained to them.

Only 31.8% of those with severe distress were currently seeing a mental health practitioner (Table 3). Of those with severe distress not seeing a mental health professional (*n* = 75), 29 (39%) were taking antidepressants.

**Table 3:** Mental health engagement by distress level

		No distress (*n* = 369)	Mild distress (*n* = 135)	Moderate distress (*n* = 96)	Severe distress (*n* = 110)
Are you seeing anyone currently for any mental health issues/stress?	Yes	29 (7.9)	28 (20.7)	18 (18.8)	35 (31.8)*

* *P* < 0.001.

## Discussion

The Australian IBD Standards^12^ detail the optimal quality of care in IBD; however, there are no studies examining how real‐life health care compares to the Standards. The present study is the largest survey of mental health needs, attitudes, and access to mental health services in people living with IBD in Australia.^17^

There is an important gap in mental health services for IBD patients in Australia: Only 12% of respondents had access to psychologists, while nearly 50% of respondents reported significant psychological distress. A previous Australian study^14^ using the same measure of distress (K10) (*n* = 336) documented clinically significant levels in 52% of respondents. A national audit of IBD hospital care^15^, ^16^ identified that “mental health condition” was the most common comorbid condition and affected more than a quarter of hospitalized patients. Less than a quarter of these patients received psychological support in hospital. Similarly, in this study, only one‐third of those with severe distress received mental health support. These statistics are in line with those of other western countries. In the United Kingdom, the provision of psychological support to IBD patients also remains at low levels, with only 12% of IBD services having access to clinical psychology via a defined referral pathway.^19^ The current UK IBD Standards list access to psychologists as a particular challenge and area for improvement.^20^ While poor access to mental health professionals is an obvious contributor to the low uptake of mental health services by people with IBD, it is presently unclear why those at high risk or with actual high severity of mental illness do not seek help, and more studies are needed in this area.^14^

Of note, only 16% of this study's respondents were asked about their mental health by their gastroenterologist or IBD nurse, and 56% of those not asked would have liked to be asked. For those who reported distress, 74% would have liked to be asked about their mental health by their gastroenterology team. While gastroenterologists and IBD nurses are not mental health providers, the early detection of symptoms of anxiety and depression is essential to prevent the progression of mental illness and to reduce the risk of suicide. Of note, while good‐quality data on suicide in IBD in Australia are not available, a large‐scale American study (*n* = 2 325 226) showed that 27% of people with IBD and depression reported suicidal ideation *versus* 12% of those without depression.^21^ A recent meta‐analysis indicated that people with IBD may have increased risk of suicide.^22^ Psychological screening is an Australian standard of care for all people with IBD.^12^ Asking about mental health when a patient seeks help for IBD is an opportunity to identify and address distress. The integrated biopsychosocial model of health care, with regular mental health screening and good access to mental health professionals in gastroenterology clinics, is likely to improve patient outcomes and reduce health‐care costs.^23^, ^24^, ^25^, ^26^, ^27^, ^28^

Furthermore, in the present survey, psychological distress was associated with several important clinical parameters: active disease, hospitalization, presence of fistulas, perianal disease, pain, and fatigue and the current use of steroids, opioids, and psychotropics. This is in line with the findings of previous studies showing that poor mental health is associated with disease flares and a generally poorer IBD prognosis.^7^, ^8^ Poor mental health has also been previously reported as a significant predictor of hospitalization,^29^ readmission,^9^ and increased risk of surgery^10^ in those with IBD. Pain, fatigue, and opioid use are also known to be associated with poor mental health in IBD.^30^, ^31^, ^32^, ^33^ Therefore, our findings demonstrate that high rates of distress are likely to contribute to the burden of IBD at the personal and societal levels and translate into large costs of IBD care in Australia.^1^

The limitations of this survey have been previously discussed^17^ and include the following: its online nature, which precluded an objective assessment of IBD status and disease activity; a female‐predominant sample of people aged 16 years and older excluded the pediatric population and limited the generalizability of its findings; and a low participation rate from people in remote areas.

In conclusion, psychological distress is prevalent in this Australian sample of people living with IBD, but access to mental health professionals in IBD clinics is limited despite the willingness of participants to discuss mental health and the association of distress with a variety of clinical parameters such as disease activity, hospitalizations, pain, fatigue, and opioid use. An integrated biopsychosocial model of health care, with regular mental health screening and good access to mental health professionals, will help people living with IBD improve their quality of life and other outcomes. Future studies should explore why many IBD patients with severe distress do not seek help and whether access to mental health professionals is the main reason for the low uptake of mental health services.
